# Emotional salience modulates the forward-flow of memory

**DOI:** 10.1098/rsos.230926

**Published:** 2024-06-12

**Authors:** Alba Peris-Yague, Darya Frank, Robin Hellerstedt, Bryan A. Strange

**Affiliations:** ^1^ Laboratory for Clinical Neuroscience, Centro de Tecnología Biomédica, Universidad Politécnica de Madrid, IdISSC, Madrid, Spain; ^2^ PhD Program in Neuroscience, Universidad Autonóma de Madrid-Cajal Institute, Madrid 28029, Spain

**Keywords:** salience, recall, emotion, episodic memory

## Abstract

As we navigate our day-to-day lives, we regularly adapt our behaviour according to what we predict may happen next in a given context. When an unexpected event occurs, our predictions about the world are disrupted and must be updated. Unexpected, isolated events, particularly with high emotionality, are also better recalled. In the present work, we investigated how oddballs affect recall dynamics. Seventy young, healthy participants encoded word lists containing either emotional or perceptual oddballs at varying stimulus onset asynchronies followed by free recall. It is well established that after recalling an item, we have a higher probability of recalling items encoded nearby, particularly those that were encoded after the item was recalled, a phenomenon known as forward contiguity of recall. We tested how novelty (oddballs versus control words) modulated forward contiguity as a function of salience type (emotional versus perceptual). The present results provide empirical evidence of forward contiguity modulation selectively by emotional salience and suggest that recall patterns after presenting emotional and perceptual oddballs are mediated by different mechanisms.

## 1. Introduction

As we navigate our day-to-day lives, we continuously adapt behaviour to what we believe will happen next in a specific context [1]. However, what if something unexpected and emotional, like witnessing a robbery, occurs? These isolated events tend to be better recalled than neutral ones in a continuous stream and are also known to affect memory for nearby events. Research has focused on understanding how these unexpected emotional events affect memory. However, less is known about how emotion modulates recall organization, affecting memory for nearby events.

Salient, oddball stimuli that deviate from the prevailing context typically show a mnemonic enhancement, a phenomenon known as the Von Restorff effect [2–4], which has been reported across item modalities [5] and at different deviance attributes [6]. In the case of emotionally salient items, the mnemonic enhancement can be accompanied by an anterograde [7] and/or retrograde [8] amnesic effect for neutral stimuli presented immediately after [9,10] or before the emotional oddball. However, there have been mixed findings regarding these properties [11] as they are modulated by task [12], stimulus onset asynchrony (SOA) [11], retention intervals and arousal characteristics of the items [11,13] and the priority of the to-be-encoded stimuli [13].

Furthermore, peri-oddball effects (i.e. for items surrounding the oddballs) have been proposed to occur as an encoding disruption of the item preceding the emotional stimulus at the synaptic and/or systems level [8,14]. Others, however, have proposed that retrograde amnesic effects in free recall could be due to item inaccessibility at retrieval, which can disappear by cueing recall [15]. This recall failure for items presented before the oddball at encoding could arise due to the likely recall of the oddball and subsequent recall transitions occurring in the forward direction (i.e. of items presented after the oddball at encoding).

Free recall dynamics and, particularly, inter-item organization can be studied using the quantitative method of conditional response probability (CRP) [16]. CRP quantifies, under the condition that item *x* is immediately followed by item *y* during encoding, the probability of recalling item *y* if *x* is recalled [16]. CRP in free recall is characterized by the generalizable findings that (i) recall transitions are more likely to be among items contiguous at encoding and (ii) to occur in the forward direction [16], which could be due to the strengthening of inter-item associations and their shared context. At retrieval, a recalled item serves as a contextual cue for the recall of related items [17]. These findings have served to develop computational models of memory [18], in which temporal context (items encoded nearby) and source context (source item’s encoding features and characteristics resulting, for example, from encoding operations performed during a given task) influence item encoding and recall dynamics. More recent computational models have included an emotionality factor [19] and developed computational models to better model memory effects in emotional disorders [20].

A strong contextual change produced, for example, by the presentation of an unexpected, oddball stimulus, is predicted to evoke a contextual item association shift from the oddball’s appearance onwards and, therefore, for upcoming items to be encoded in an updated or modulated context. Brain correlates of this context-updating are considered to be reflected by the P300 event-related potential component in electroencephalography (EEG) studies; if a new, or unexpected, stimulus is detected in a stream of stimuli it evokes a P300 potential [21].

In the present study, we tested the hypothesis that recall of emotional (aversive in content) and perceptual (presented in a different font) oddballs prompts an update in the source context, thereby promoting a contextual update and, thus, a stronger contextual binding with the updated context for items encoded after the oddballs. We predicted this would result in increased forward-contiguity for items encoded after oddballs. Lastly, we hypothesized that oddballs would be recalled early on and that transitions from emotional oddballs would be enhanced, which would correlate with an oddball-induced retrograde amnesic effect. However, we acknowledge that some studies have reported weaker anterograde amnesic effects after emotional oddballs [9,10], but this has not been observed in the task employed here [8,22]. Strange *et al*. [8] showed that oddballs elicited a retrograde amnesic effect that spanned for two items preceding oddballs. As items in this previous paradigm were presented every 3 s, it remained unclear whether the amnesic effects were due to timing effects or item capacity. Therefore, in the present study, we aimed to investigate whether the induced retrograde amnesia spanned to 2 items or 6 s by varying SOAs between stimuli.

## 2. Methods

### 2.1. Subjects

Seventy healthy right-handed native Spanish-speaking subjects took part in this study (35 males, 35 females (age range, 18–32 yr; mean age, 22.5)). All subjects gave informed consent and did not have neurological or psychiatric history. The sample size was determined from the reported emotional retrograde amnesic effects in fig. 2*a* in Strange *et al*. [8]. Specifically, the reported effects had an effect size of dz = 1.69, which at 95% power reflected a need for a sample size of *n* = 7. Given in our current paradigm, we included an SOA manipulation and had both emotional and perceptual lists, which resulted in a total required sample size of *n* = 70 participants.

### 2.2. Task

**Figure 1.**
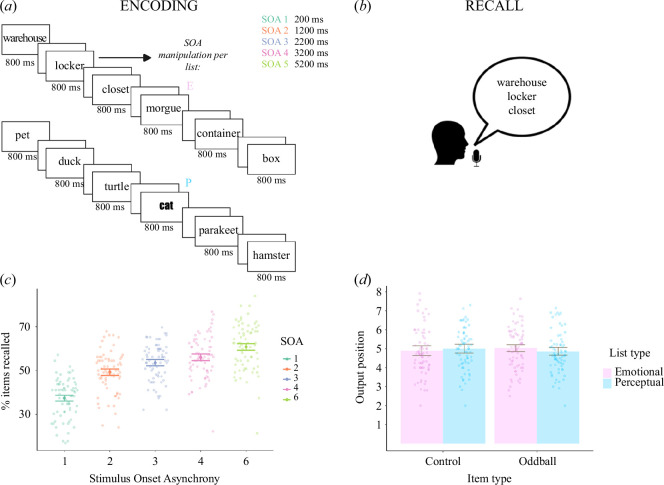
Memory task and total recall performance for list types. (*a*) Example of items used in the task where E and P are the Emotional and Perceptual oddballs. Each word was presented for 800 ms, followed by a blank screen where presentation timings varied depending on the SOA, which was kept constant within a list. (*b*) Example of forward-contiguity in recall where items near each other are more likely to be recalled and more so in the forwards direction. (*c*) The number of items recalled increased as SOA augmented. Percent of items recalled per list is plotted for each SOA; bars show subjects’ mean ± 95% confidence intervals. Jittered points show single subject mean recall. (*d*) Output position of both oddballs for each SOA and controls, matched by input position. Bars show subjects’ mean ± 95% confidence intervals. Jittered points show single subject mean output position.

The present paradigm was the translated version of previously normed stimuli in [6,8,22,23], which constructed the semantically related lists of nouns using the Edinburgh associative thesaurus [6]. We did not explicitly control for familiarity of words or word length. Lists from Strange *et al*. [8] were translated into Spanish (electronic supplementary material, tables S6 and S7). Subjects were presented with 40 lists of 14 nouns, with the words ‘New List’ presented between lists. To control semantic effects, 13 of the nouns were of the same semantic category (e.g. animals, occupations), emotionally neutral, and were presented in the same font (referred to as standard nouns). To set the context, the first five nouns in each list were always standard (i.e. not oddballs). Twenty lists contained an emotional oddball, aversive in content but of the same semantic category and perceptually identical to control nouns. Control nouns were pre-selected items within each list to be later used as a recall reference, i.e. to calculate recall of the oddballs with respect to a pre-selected non-oddball item and were, at least, three serial positions apart from the oddballs. The remaining 20 lists contained a perceptual oddball. Oddballs were randomly allocated to the 7, 8, 9, 11 or 12th serial position, to maximize list position distance between oddballs and control nouns, permitting at least two serial positions following an oddball (if presented at serial position 12). All nouns were presented in Times font, except for perceptual oddballs, which were presented in 20 different fonts. The order of the oddball list type was random. Nouns were presented visually in lowercase for 800 ms. Subjects made push-button responses to indicate whether the first letter in each noun contained an enclosed space (shallow encoding task). To investigate how the presentation rate of items affected peri-oddball amnesic effects, we varied the rate of stimulus presentation randomly at a SOA of 1, 2, 3, 4, or 6 s. In Tulving’s [24] original paradigm, retrograde amnesic effects were reported for words presented at a rate of 1 but not 2 s and [12] reported memory enhancement for items preceding emotionally arousing images at a presentation rate of 4 s. Lastly, to determine whether the retrograde amnesic effect reported by Strange *et al*. [8] spanned to 2 items or 6 s and whether SOA influenced oddball effects on CRP, SOAs of 3 and 4 s were included in the experimental design. Thus, for each of the 20 lists for each oddball type, 4 of these lists were presented at a given SOA. Subjects were informed of the presentation rate in each forthcoming list by presenting the SOA under the ‘New List’ marker (figure 1*a*). Control nouns, like the oddballs, could not occur within the first five nouns of each list and were at least three serial positions apart from oddball nouns to control for peri-oddball effects.

The presentation of each list was followed immediately by a 30 s distractor task, during which subjects were instructed to count backwards in steps of 3 (out loud) from a number presented on the screen followed by instructions to recall the words presented in the preceding list.

To test, *a posteriori*, for semantic relatedness and emotional valence a separate online task was conducted on independent experimental groups. Participants were presented with an online questionnaire that contained all 40 experimental lists. To test for semantic relatedness, each list (with 14 items) was presented at a time and participants (*n* = 14) were instructed to carefully read the whole list and then rate how each item was related to the rest (with 1 being not very and 10 being very much related). Emotional valence ratings (*n* = 15) were obtained by another independent raters sample (*n* = 15) who carefully rated each item on a scale from −6 (negative value) to 6 (positive value).

### 2.3. Statistical analyses

Analyses were conducted using MATLAB (R2019b, The MathWorks, Inc). Statistical analyses and figures were conducted in Rstudio (version 1.3.1093) and JASP (Jasp Team, 2021). Statistical analyses were conducted using a within-subjects, repeated measures (RM) ANOVA as each subject was presented with both emotional and perceptual lists. Factors included in each analysis are specified where relevant. Post-hoc *t*-tests were FDR-corrected and effect sizes were calculated with partial eta squared (*η*_p_^2^) or Cohen’s *d*. Bayesian statistics were conducted on JASP as a Bayesian RM-ANOVA comparing effects across all models. We used default prior options established on JASP (*r* = 0.5 for the fixed effects).

CRP analyses were conducted using original and modified versions of the Behavioural Toolbox for MATLAB R2019b (http://memory.psych.upenn.edu/Behavioral_toolbox). *Lag* refers to the word-distance to an item at encoding; all analyses and visualization were performed on lags ± 5 as previously reported [16]. *Backwards* versus *forwards* refer to words presented before or after a specific item at encoding, analogous to negative and positive lags, respectively. In the present modified version of the analysis, a lag of 0 refers to recalling the oddball or control word, whereas usually, in CRP analyses, a lag of 0 is an item that could have been encoded anywhere in the list. In this manuscript, the terms *lags* and *transitions* are used interchangeably.

## 3. Results

Recall performance improved with increasing SOA (figure 1*c*). There was an oddball recall enhancement compared with selected control items presented in the same serial encoding position in a different list of the same category (electronic supplementary material, figure S1*a*). However, the current, Spanish version of this task did not elicit an emotion-induced retrograde amnesic effect as previously reported, which was confirmed by strong evidence for a null effect of peri-oddball amnesic effects using Bayesian statistics (see electronic supplementary materials, results and figure S1*b*,c, table S2). Due to the lack of retrograde amnesic effects, we conducted further rating tasks in an independent sample of participants, which confirmed emotional negative valence but also showed a decrease in semantic relatedness in emotional lists compared with perceptual ones (electronic supplementary material, tables S6 and S7). We ran generalized linear mixed-effects models to test firstly whether potential amnesic effects were modulated by SOA while accounting for semantic relatedness of list items (electronic supplementary material, table S8), and secondly whether memory for item-1 words was modulated by semantic similarity (electronic supplementary material, table S9).

Participants tend to adopt different recall strategies as a function of list length. Specifically, in shorter lists, e.g. with seven or less items, participants tend to start recall at the beginning of a list, whereas in longer lists, they do so towards the end of the list [25,26]. Here, we consistently found that items encoded at early serial positions had a higher probability of first recall (electronic supplementary material, figure S4).

### 3.1. CRPs showed an enhanced forward contiguity effect after recalling emotional oddballs

To comprehensively evaluate recall properties, we first evaluated oddball recall position, hypothesizing that oddballs would be recalled early in the serial recall order. RM-ANOVA with novelty (control, oddball) and saliency type (emotional, perceptual) as factors showed that this was not, however, the case (figure 1*d*). Neither emotional nor perceptual oddballs were recalled early compared with input-matched control items (figure 1*d*, no significant main effect (m.e.) of saliency type (emotional, perceptual) *F*(1,68) = 0.006, *p* = 0.94, *η*_p_^2^ = 0.00, no m.e. of item recall position (control, oddball) *F*(1,68) = 0.078, *p* = 0.78, *η*_p_^2^ = 0.00, nor a significant interaction *F*(1,68) = 2.07, *p* = 0.155, *η*_p_^2^ = 0.00).

**Figure 2.**
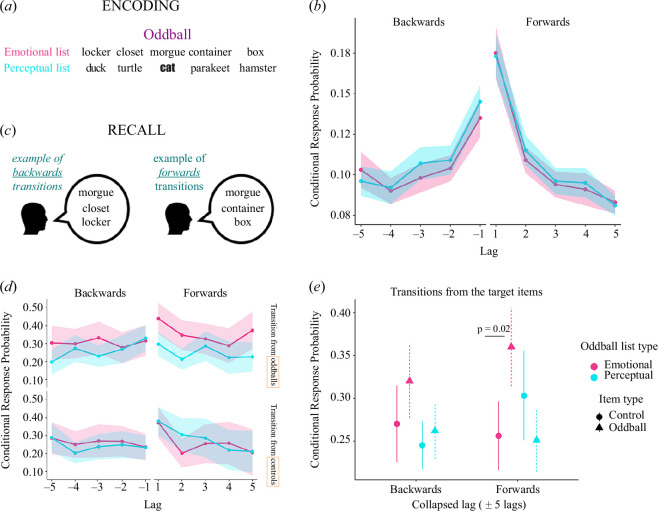
Transitions from emotional oddballs are enhanced compared with controls. (*a*) Example of encoding emotional and perceptual lists. (*b*) Preserved forward contiguity across both list types. (*c*) Example of backwards transitions where the oddball would be recalled and followed by the recall of items preceding it at encoding and forward transitions whereby the oddball would be recalled and followed by items encoded after the oddball. (*d*) CRP curves showing enhanced forward transitions from emotional oddballs compared with pre-selected control items (mean ± confidence intervals). (*e*) CRP values (mean ± 5 lags) showing increased CRP in forward transitions from emotional oddballs compared with control items.

Next, we tested whether the presentation of an oddball would provoke a shift in the encoding context which would lead to items encoded after the oddballs to be studied in an updated context and to be more strongly bound to the oddballs. To do so, we investigated, with CRP curves, whether the presence of an oddball affects forward recall transitions: hypothesizing that there would be an enhancement in transitions, particularly from emotional oddballs, which would, in turn, explain a potential retrograde amnesic effect. Indeed, the present results showed an enhancement in forward transitions from emotional oddballs (figure 2*d,e*).

Forward transitions, with saliency type (emotional, perceptual) and novelty (oddball, controls) showed a significant novelty x salience interaction (*F*(1,69) = 12.21, *p* < 0.001, *η*_p_^2^ = 0.15). Transitions from emotional oddballs were enhanced compared with transitions from pre-selected control items in emotional lists (figure 2*d,e*) t(69) = −3.23, *p* < 0.005, *Cohen’s d* = −0.386) while transitions from perceptual oddballs did not differ from pre-selected control items in perceptual lists (t(69) = 1.73, *p* = 0.09, *Cohen’s d* = 0.206). There was no significant m.e. of saliency type (emotional, perceptual) (*F*(1,69) = 2.675, *p* = 0.11, *η*_p_^2^ = 0.037) or novelty (oddball, control) (*F*(1,69) = 1.36, *p* = 0.25, *η*_p_^2^ = 0.019). For completion, we tested that this enhancement was not present in backwards transitions. A two-way RM-ANOVA, in backwards transitions, between saliency type (emotional, perceptual) and novelty (oddball, control noun) showed a significant m.e. of saliency type (F(1,69) = 3.992, *p* = 0.050, *η*_p_^2^ = 0.055) and novelty (F(1,69) = 4.65, *p* = 0.035, *η*_p_^2^ = 0.06) but no significant oddball saliency type x novelty interaction (F(1,69)=0.98, *p* = 0.33, *η*_p_^2^ = 0.014).

These results indicate that CRP values in backwards transitions (negative lags) were higher for emotional than perceptual lists as well as higher for transitions from oddball items compared with transitions from controls in the same lists. However, forward transitions (positive lags) showed an enhancement in CRP values from emotional oddballs relative to control items and perceptual oddballs. Although we did not observe a retrograde amnesic effect in the present task (electronic supplementary material, figure S1*b*), for completeness, we evaluated whether the *forward flow* from emotional oddballs correlated with recall of items preceding emotional oddballs. We had initially hypothesized there would be a presence of retrograde amnesic effects, which could potentially be explained by a stronger tendency to transition in the forward direction from oddballs in a recall. However, we did not find this to be the case (electronic supplementary material, figure S3). Lastly, across all stimuli in both types of lists, recall transitions preserved forward-contiguity overall (figure 2*b*) and across SOAs (electronic supplementary material, figure S2) as typically reported in free recall tasks.

## 4. Discussion

We used a word oddball paradigm containing emotional and perceptual oddballs to investigate recall organization after unexpected events. Overall, both emotional and perceptual oddballs were better recalled than all other items. Previous research using similar paradigms had reported peri-oddball amnesic effects, often resulting in anterograde and/or retrograde amnesia for items presented after/before the oddballs [11]. However, such effects were not observed in the current version of the paradigm.

We calculated CRP curves on recalled items from an oddball paradigm that employed word lists containing either an emotional or perceptual oddball. Considering all words presented at encoding, forward-contiguity, a key property of free recall [16], was preserved. We further looked at transitions to and from the oddballs to evaluate whether these core properties remained present. Interestingly, while we found that contiguity was maintained for both oddball types and transitions to and from oddballs, there were enhanced CRP forward transitions from emotional oddballs—relative to control nouns—which were not present in transitions from perceptual oddballs. These effects could represent an underlying stronger binding of temporal order between emotional items and succeeding items due to a contextual update.

We did not find support for the hypothesis that oddballs are recalled earlier than matched input position control items [19] (figure 1*d*). We found a consistent tendency for a higher probability of first recall of items encoded early-on although participants encoded long lists of items, contrary to what has been reported by [25,26]. We speculate this may be because of the presence of a distractor task before recall, as the overall reported oddball effect was similar to previous work with similar list-length tasks [6,8,23,27]. Our current empirical findings suggest that emotional and perceptual oddballs modulate memory-related CRP differently, and these differences could inform the future development of computational models of memory. The neurobiological processes underlying emotional and perceptual salience have also been dissociated in studies with pharmacological manipulations; while recall in the former was modulated by cortisol levels [28,29], the adrenergic system and focal amygdala lesions, the latter was not [8]. The present task in combination with pharmacological manipulations of the beta-adrenergic system could provide insight into the biological mechanisms behind the CRP lag contiguity property of memory recall and its modulation by emotional salience.

Previous studies using oddball paradigms found a mnemonic enhancement for oddballs accompanied by a retrograde amnesic effect [8,9,11,30]. Given that we found a forward-flow enhancement in transitions from emotional oddballs, we hypothesized that it would serve as an anchor to move forwards in recall and, thus, explain the retrograde amnesic effect. However, we did not find such a significant correlation, most likely because of the lack of retrograde amnesic effects in the present task. The lack of retrograde effects is unlikely to reflect list length, as E-1 memory deficits have previously been observed with 14-item lists [8]. We speculate that a lack of emotion-induced retrograde amnesia, despite a memory enhancement for E nouns of similar magnitude to previous studies [8,22,23,27], may be attributable to some aspect of the Spanish language used in the current task. A potential explanation for this could be that the translated items used in the current paradigm did not elicit the same levels of arousal as items in previous experiments [31] or that in this paradigm, the peri-oddball amnesic effects were modulated by the inclusion of a distractor task as previously shown [8,11,32]. How emotion modulates the perception of the spatiotemporal emotional and neutral items has been the focus of experiments using temporal order paradigms. These have yielded mixed findings regarding the effects of emotion on temporal order and highlighted the influence of encoding and retrieval tasks on different results [33]. Recently, Clewett & McClay [34] found a favorable carryover emotional effect to benefit within-events binding and Riegel *et al*. [35] reported better temporal order for object pairs spanning emotional items. In more naturalistic movie settings, although temporal clustering remained unaltered, Dev *et al*. [36] found that emotion improved temporal order. Future studies could combine free recall paradigms with a temporal order memory task to better understand emotional carryover effects.

In the present task, we found that emotional lists had decreased semantic relatedness compared with perceptual lists; however, if decreased semantic associations within emotional lists were strongly influencing the present results, we would have expected to see diminished CRP curves in emotional lists. This, however, was not the case as we observed an enhancement of CRP values from emotional oddballs. In the present study, we applied CRP curve analysis on a free recall paradigm to investigate emotional and perceptual salience. Oddballs were not retrieved earlier than control words. We found an enhancement in recall transitions from emotional oddballs, which was not present in transitions from perceptual oddballs. These results show a dissociation in emotional and perceptual salience at recall and provide empirical evidence that could be used to update computational models of emotional memory and the von Restorff effect.

## Data Availability

Raw data are available at [37] as well as here (with analysis code) [38]. Supplementary material is available online [39].
